# A Comparative Study of Clinicopathological Features between Chronic Cholecystitis Patients with and without *Helicobacter pylori* Infection in Gallbladder Mucosa

**DOI:** 10.1371/journal.pone.0070265

**Published:** 2013-07-30

**Authors:** Di Zhou, Wen-bin Guan, Jian-dong Wang, Yong Zhang, Wei Gong, Zhi-wei Quan

**Affiliations:** 1 Department of General Surgery, Xinhua Hospital, Shanghai JiaoTong University, School of Medicine, Shanghai, China; 2 Department of Pathology, Xinhua Hospital, Shanghai JiaoTong University, School of Medicine, Shanghai, China; Institut Pasteur Paris, France

## Abstract

**Background:**

*Helicobacter pylori* has been isolated from 10%–20% of human chronic cholecystitis specimens but the characteristics of “*Helicobacter pylori* positive cholecystitis” remains unclear. This study aims to compare the clinicopathological features between chronic cholecystitis patients with and without *Helicobacter pylori* infection in gallbladder mucosa.

**Methods:**

Three hundred and twenty-six chronic cholecystitis patients were divided into two groups according to whether *Helicobacter pylori* could be detected by culture, staining or PCR for *Helicobacter* 16s rRNA gene in gallbladder mucosa. Positive samples were sequenced for *Helicobacter pylori*-specific identification. Clinical parameters as well as pathological characteristics including some premalignant lesions and the expression levels of iNOS and ROS in gallbladder were compared between the two groups.

**Results:**

*Helicobacter pylori* infection in gallbladder mucosa was detected in 20.55% of cholecystitis patients. These patients had a higher prevalence of acid regurgitation symptoms (*p* = 0.001), more histories of chronic gastritis (*p* = 0.005), gastric ulcer (*p* = 0.042), duodenal ulcer (*p* = 0.026) and higher presence of *Helicobacter pylori* in the stomach as compared to patients without *Helicobacter pylori* infection in the gallbladder mucosa. *Helicobacter pylori* 16s rRNA in gallbladder and gastric-duodenal mucosa from the same individual patient had identical sequences. Also, higher incidences of adenomyomatosis (*p* = 0.012), metaplasia (*p* = 0.022) and higher enhanced expressions of iNOS and ROS were detected in *Helicobacter pylori* infected gallbladder mucosa (*p*<0.05).

**Conclusions:**

*Helicobacter pylori* infection in gallbladder mucosa is strongly associated with *Helicobacter pylori* existed in stomach. *Helicobacter pylori* is also correlated with gallbladder premalignant lesions including metaplasia and adenomyomatosis. The potential mechanism might be related with higher ROS/RNS production but needs further investigation.

## Introduction

Chronic cholecystitis is one of the most prevalent diseases requiring surgical intervention. In China, more than 90% of the cholecystitis cases are claimed to be caused by symptomatic cholelithiasis, the incidence of which is approximately 10% of the adult population. [1], [2] Histologically, chronic cholecystitis presents a large range of related inflammatory epithelial changes including mononuclear infiltrate, fibrosis, thickness of muscular layer, dysplasia, hyperplasia and metaplasia-the last three have been considered as premalignant lesions. [3]–[5].

The causes of chronic cholecystitis still remain unclear. Recently, many findings obtained from microbiological studies suggest that bacterial infection in biliary system might play a role. Our previous meta-analysis demonstrated that *Helicobacter pylori* (*H.pylori*) in human biliary system was correlated with chronic cholecystitis, especially in the regions with higher prevalence of this infectious agent such as South Asia, East Asia and Latin America. [6] Evidences supporting the association between *H.pylori* infection and chronic cholecystitis could be found by using direct culture or staining of *H.pylori* in gallbladder tissues as well as indirect techniques such as PCR, ELISA and serology for detecting *H.pylori*-specific genes or antibodies.[7]–[9] The positive rate of *H.pylori* in gallbladder is reported to be 10%–20% by culture. [10].

*H.pylori* can induce oxidative stress through producing reactive oxygen species (ROS) and reactive nitrogen species (RNS), which are considered to be the important causes of chronic inflammation, ulcer and canceration of the stomach. [11] In *H. pylori*-infected stomach, possible sources of ROS/RNS include neutrophils, vascular endothelial cells, gastric mucosal cells, and *H. pylori* itself. One of the most important pathways of *H.pylori*-induced RNS is mediated by overproduction of endogenous synthesis nitric oxide (NO) through inducible NO synthase (iNOS) expression. [12] In benign inflammatory and malignant gallbladder diseases, ROS and iNOS also play an important role. [13] However, in biliary system, the correlation between *H.pylori* and ROS/RNS production still needs further investigation.

Two-thirds of the world population is infected with *H.pylori*. [14] The findings of *H.pylori* in biliary tract implicated that the stomach might not be the only arena of activity of this agent. However, few studies by far have specifically assessed the characteristics of “*Helicobacter pylori* positive cholecystitis”. Therefore, this study aims to compare the clinicopathological features between chronic cholecystitis patients with and without *Helicobacter pylori* infection in gallbladder mucosa.

## Materials and Methods

### Patients

Of 378 patients who underwent cholecystectomy in Department of General Surgery, Xinhua Hospital from December 2011 to July 2012, three hundred and twenty-six patients (97 males and 229 females, aged 21–87 years) who fulfilled the pathological criteria of chronic cholecystitis were enrolled in this study. The exclusion criteria were: (1) patients with history of hepato-biliary or pancreatic surgery which changed the normal structure and function of the biliary system, (2) patients who had previously received standard triple therapy for *H. pylori* eradication, (3) patients who had taken antibiotics or proton pump inhibitors 4–6 weeks prior to cholecystectomy. According to whether *H. pylori* was detected positive in gallbladder mucosa, patients were divided into two groups. The study protocol was approved by the Ethics Committee of Shanghai JiaoTong University, School of Medicine and signed informed consent was obtained from all the patients.

### Gastroscopy

Before or after cholecystectomy, all patients enrolled in this study received gastroscopy with biopsy in order to clarify the infection status of *H. pylori* in their stomach. Gastroscopy was performed with video endoscopes that worked in high-resolution, white light mode and AFI mode (EVIS-FQ260Z; Olympus Medical Systems Co. Ltd, Tokyo, Japan). Two biopsy specimens were taken at each site from the greater curvature of the antrum, and the greater and lesser curvature of the corpus. Each of the two specimens from the above parts of the stomach were used respectively for culture and Warthin-Starry Staining of *H. pylori*.

### Cholecystectomy and Gallbladder Biopsy

Laparoscopic cholecystectomy was performed by a single surgeon using a standardized 4-port technique (Laparoscopic Device, KARL STORZ GmbH, Tuttlingen, Germany). Two biopsy specimens were taken aseptically at each site from the fundus, body and neck of the gallbladder. Each of the two specimens from the above parts of the gallbladder were used respectively for culture and Warthin-Starry Staining of *H. pylori*.

The stomach and gallbladder specimens were aseptically transferred to the microbiology laboratory immediately after gastroscopy or cholecystectomy.

### Verification of *H. pylori* Infection in Gallbladder and Stomach

The presence of *H. pylori* in gastric or gallbladder mucosa was determined by either positive culture, Warthin-Starry Staining or positive nest PCR for specific 16s rRNA of this bacterium. At least one positive test was regarded as confirmation of infection of this agent in gallbladder or gastric mucosa.

### Culture of *H. pylori*

The gallbladder and gastric mucosa specimens were inoculated onto sterile plates containing endo agar and Brucella agar supplemented with 5% horse blood (Becton, Dickinson and Company, Sparks, Maryland, USA) for nonspecific bacterial and *Campylobacter* cultures, respectively. For isolation of *H.pylori*, we prepared specific media containing BHI agar supplemented with 7% sheep blood, 0.4% IsoVitaleX (Becton, Dickinson and Company, Sparks, Maryland, USA) and Skirrow-selective supplements (Oxoid Limited, Thermo Fisher Scientific, Hampshire, UK). *H.pylori* was incubated at a microaerophilic condition (5% O_2_, 10% CO_2_, 85% N_2_) for 3–7 days at 37°C. Bacteria colonies were examined with biochemical tests as well as microscopy for conformation. *H. pylori* colony was identified as showing the typical white, pin-point and transparent morphology. Oxidase and fast urease activity were also performed at colonies grown on *H. pylori* specific media. Bacterium with typical morphology, positive oxidase and urease activity were verified as *H. pylori*.

### Warthin-Starry Staining of *H. pylori*

Warthin-Starry Staining was performed using the specific kit (Diagnostic BioSystems, Pleasanton, California, USA). Four-micrometer thick paraffin sections of gallbladder and gastric mucosa tissues from each patient were backed for 1 h at 60°C. After dewaxing and re-hydration, sections were incubated at 56°C for 1 h in 1% silver nitrate buffer in the dark box and then dipped in developer solution and stained for 5–8 min. Finally, sections were dehydrated with 100% alcohol, cleared with xylene. *H. pylori* was identified as stained into buffy or black color in a light yellow background under microscope with oil immersion lens (×1000). The results were also determined independently by the above two pathologists. Warthin-Starry staining and *H. pylori* culture were blindly assayed for all the specimens from each patient.

### PCR for *Helicobacter* 16s rRNA Gene

The DNA extracts were prepared from the paraffin specimens as the kit (TIAN amp Micro DNA kit, TIANGEN Biotech, Beijing, China) instructed. A semi-nested PCR assay specific for *Helicobacter* 16s rRNA gene (16s rDNA) was amplified as previously described [15], using primers 1F (5′CTATGACGGGTATCCGGC3′), 1R (5′CTCACGACACGAGCTGAC3′) and 2R (5′TCGCCTTCGCAATGAGTATT3′). Primers 1F and 1R were used in the first step, whereas primers 1F and 2R were used in the second step. The PCR reaction mixture contained 1 µl of 10 µM each primer (1F and 1R), 2 µL of 10 mM dNTP, 1×PCR reaction buffer, 2.5 mM MgCl2, 0.05% casein, 0.05% formamid, 1.25 U rTaq DNA polymerase (Takara, Dalian, China), and 2 µL extracted DNA as templates. The amplification conditions for the first step were 94°C for 2 min; 30 cycles of 94°C for 30 s, 55°C for 30 s, and 72°C for 30 s; and finally 72°C for 10 min. The reaction mixture of the second step (25 µL) contained 0.5 µl of 10 µM each primer each primer (1F and 2R), 0.2 mmol/L each dNTP, 1×PCR reaction buffer, 2.5 mmol/L MgCl2, 1.0 U ExTaq DNA polymerase (Takara, Dalian, China), and 2 µL 10×diluted PCR product from the first step. The 416-bp PCR products were visualized by 1.2% agarose gel electrophoresis.

### Sequence Analysis

The PCR products were subsequently ligated into pMD-19T simple vector (Takara, Dalian, China) for sequencing. The product was sequenced after PCR amplification and double enzyme digestion. The sequences were compared with the 16S rRNA gene sequences of the strains that have been registered in the GenBank database. Products of the sequence reaction were aligned and the closest homologous sequence was identified by Standard Nucleotide BLAST analysis tool on the internet (BLASTn, http://blast.ncbi.nlm. nih.gov).

### Histological Analysis of Chronic Cholecystitis

Gallbladder specimens were fixed in 10% buffered formalin and embedded in paraffin. Four micrometer-thick sections were cut and stained with hematoxylin-eosin (HE). Sample slides were examined independently by two attending pathologists who specialized in biliary diseases and in the case of discrepancy, the decision was made by discussion or in consultation with a third experienced pathologist. Chronic inflammation was diagnosed in the presence of a predominantly mononuclear inflammatory infiltrate, fibrosis, or metaplastic changes. A scoring system proposed by Barcia JJ [16] was used to semi-quantitatively assess the histological changes of chronic cholecystitis (Table 1).

**Table 1:** Definitions of Pathological Changes of Chronic Cholecystitis.

Pathological Changes of Chronic Cholecystitis	Definition
Inflammatory mononuclear infiltrate	
Mild	Diffuse, ≤10 inflammatory cells per HPF in any layer
Moderate	Diffuse, between 11 to 30 cells per HPF
Severe	Diffuse, more than 31 cells per HPF or follicular
Degree of fibrosis	
Mild	Uneven collagen deposition in ≤20% of material
Moderate	Uneven collagen deposition in 21% to 70% of material
Severe	Uneven collagen or lamellar fibroplasia in ≥71% of material
Thickness of the muscular layer	
Mild	Less than one third of the whole thickness
Moderate	One third to two thirds of the wall
Severe	More than two thirds of the wall thickness
Addipose tissue deposition	
Mild	Up to 10% of the material
Moderate	11% to 60% of the material
Severe	More than 60% of the material
Degree of hyperplasia	
Diffuse	≥70% of the whole sections
Focal	<70% of the whole sections
Degree of dysplasia	
Low-grade	Resemble tubular adenomas of the colon without intestinal metaplasia
High-grade	Markedly pleomorphic nuclei and/or prominent nucleoli
Metaplasia	
Pyloric type	Structures similar to the pyloric glands in the lamina propria
Intestinal type	Goblet cells and enterocitlike cells
Gastric surface type	Epithelial cells of gallbladder mucosa replaced by tall columnar cells with abundant mucin and basallylocated nuclei

HPF: high power field.

### Immunohistochemical Staining of iNOS and ROS

For iNOS and ROS detection, immunohistochemistry was performed on 4-µm thick, mounted on silane-coated slides of gallbladder mucosa tissue sections. Sections were deparaffinized and rehydrated, then washed in distilled water and 0.05 mol/L Tris buffer. After blocking the nonspecific binding sites by using Protein blocking agent (Coulter-Immunotech, Marseille, France), sections were incubated with the primary polyclonal rabbit anti-iNOS and ROS antibody (1∶200, Transduction Laboratories, kentucky, USA) at 4°C for 24 h. After sections were washed, a biotinylated immunoglobulin (anti-rabbit serum for iNOS and ROS) was applied for 30 min. Finally, all sections were incubated with the avidine-biotin-complex (ABC) with alkaline phosphatase (Vectastain, Vector Laboratories, Burlingame, California, USA). The staining was visualized with 3,3′-diaminobenzidine and hydrogen peroxide.

Semiquantitative analysis of the iNOS and ROS immunostaining was performed independently by the above two pathologists and in the case of discrepancy, the decision was made by discussion or in consultation with a third experienced pathologist. In ten randomly selected areas of the whole section, the number and percentage of positive cells were calculated for determining staining intensity and proportion of iNOS or ROS staining. A case without positive cells was considered negative. A case with less than 10% positive cells was scored 1, 10–50% was scored 2, 50–80% was scored 3 and more than 80% was scored 4. A staining intensity was classified as weak (I), moderate (II) and strong (III). A immunoreactive score was calculated as staining intensity×amount of positive cells (from lowest score 0 to highest 12) and specimen with a grade of more than 1 was defined as positive. [17].

### Statistical Analysis

The software SAS 9.13 (SAS Institute, Gary, North Carolina, USA) was used for conducting statistical analysis. Student’s t-test was performed for comparing age and BMI. The immunoreactive score of iNOS and ROS were calculated and statistically compared between the two groups using Mann-Whitney *U*-test. Chi-square test or Fischer’s exact test was used to examine the rest clinicopathological parameters. For all statistical analyses, significance levels were set at *p*<0.05.

## Results

**Figure 1:**
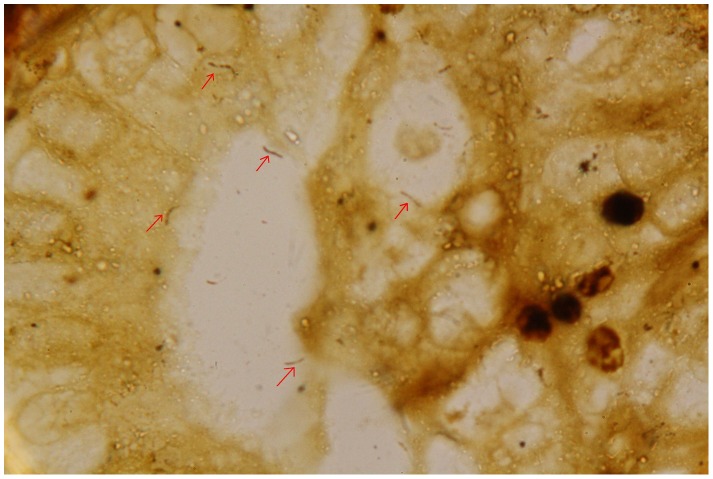
*H.pylori* infection in metaplastic gallbladder mucosa (oil immersion lens,×1000, red arrow indicates *H.pylori*).

**Figure 2:**
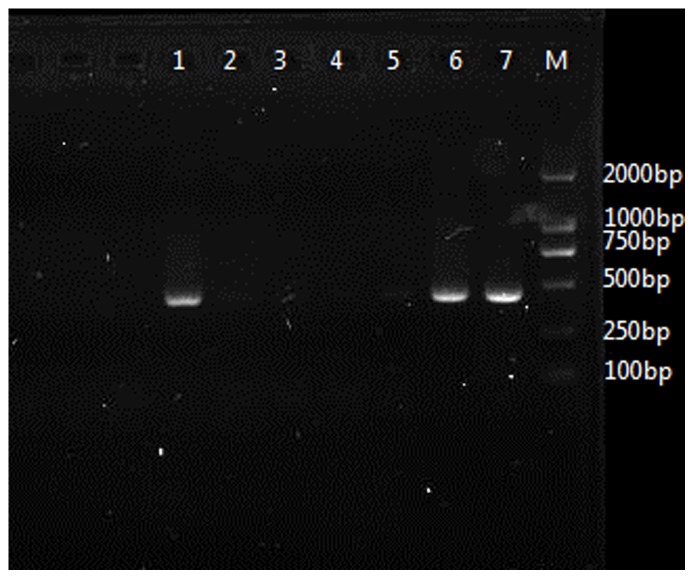
PCR products of *Helicobacter* specific 16s rRNA gene from gallbladder and gastric mucosa samples. (lanes M: step-ladder marker; 1: positive control of gastric biopsy-derived *H. pylori* DNA; 2: negative control of gastric biopsy; 3: negative 16s rRNA gene in gallbladder; 4 and 5: negative 16s rRNA gene in gallbladder and gastric mucosa acquired from one individual patient; 6 and 7: positive 16s rRNA gene in gallbladder and gastric mucosa acquired from another individual patient).

**Figure 3:**
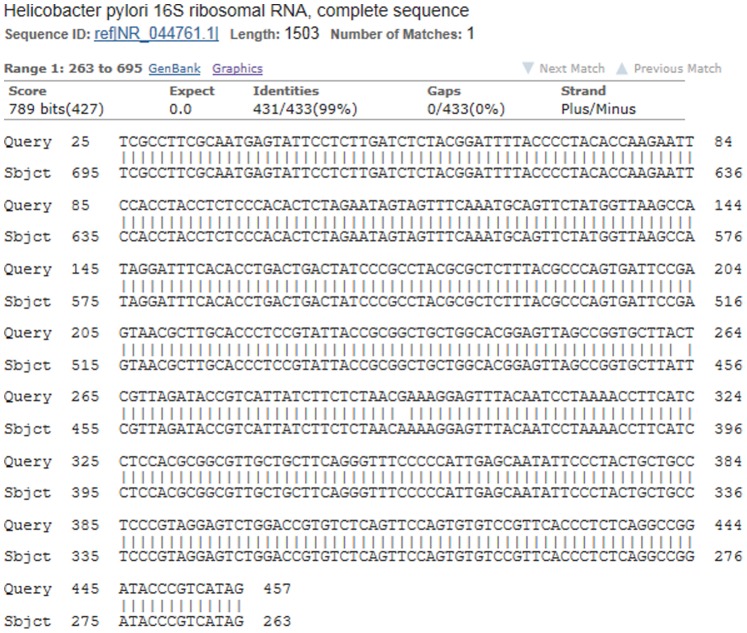
Comparison of complete sequence data of *H.* *pylori* 16s rRNA gene tested in gallbladder mucosa sample from published GenBank data: sequence ID ref|NR_044761.1|. (nucleotides 263–695 were listed).

**Figure 4:**
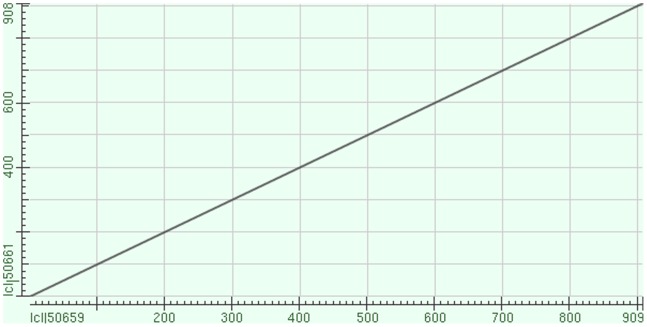
BLAST showed *H.* *pylori* 16s rRNA gene in gallbladder and gastric mucosa from the same individual patient had completely identical sequences. (sequence ID50661: H. *pylori* 16s rRNA tested in gallbladder mucosa; sequence ID50659: H. *pylori* 16s rRNA tested in gastric mucosa).

Evidence of *H. pylori* in gallbadder mucosa was demonstrated by Warthin-Starry staining in 64 (19.63%) patients and *H. pylori* colonies were identified upon culture in 55 (16.87%) patients. Among them, 52 (77.61%) patients were both positive in staining and culture. In PCR test for *Helicobacter-*16s rRNA gene, 67 (20.55%) patients were positive. From all the gallbladder specimens, only the positive samples which detected by staining or culture were positive in nest PCR test. All samples positive for first-step amplicon were also positive for the nested PCR. Finally, *H. pylori* infection in gallbladder mucosa was detected in 20.55% (n = 67) of the cholecystitis patients (Figure 1 and 2). These patients had a higher prevalence of acid regurgitation symptoms (*p* = 0.001), more histories of chronic gastritis (*p* = 0.005), gastric ulcer (*p* = 0.042), duodenal ulcer (*p* = 0.026) and a higher positive rate of *Helicobacter pylori* (*p*<0.05) in the stomach as compared to patients without *Helicobacter pylori* infection in the gallbladder (Table 2). Of the 67 patients (20.55%) who were positive in *H. pylori* 16s rRNA detection in gallbladder mucosa, amplications of 16s rRNA in their gastric or duodenal specimens were also succeed in 42 patients (62.69%). These were 30 of 45 (66.67%) patients with chronic gastritis, 7 of 11 (63.64%) patients with gastric ulcer and 5 of 8 (62.50%) patients with duodenal ulcer (Figure 2). Consequently, we check the amplified PCR products by direct sequencing and BLAST search and confirmed that each sequence was 96–99% similar to a known *H. pylori* 16s rRNA gene registered in GenBank (Figure 3). No other kinds of *Helicobacter* species such as *Helicobacter bilis*, *Helicobacter hepaticus* or *Helicobacter pullorum* could be detected by PCR. Moreover, our data also revealed that *H. pylori*-16s rRNA in gallbladder and gastric (or duodenal) mucosa acquired from the same individual patient had identical sequences (Figure 4).

**Table 2:** Clinical characteristics of *H.pylori*-positive and negative chronic cholecystitis.

Characteristics	No. of Patients n (%)		
	*H.pylori* (+) in gallbladder mucosa (n = 67)	*H.pylori* (−) in gallbladder mucosa (n = 259)	*p* value
Age (yr)	45.54±12.58	47.82±11.56	NS
Gender			
Male: Female (% Male)	19∶48(28.36)	78∶181(30.12)	NS
BMI (kg/m^2^)	23.10±2.24	22.72±1.85	NS
Symptom			
Mild Abdominal Pain	44(65.67)	149(57.53)	NS
Biliary Colic	32(47.76)	102(39.38)	NS
Loss of Appetite	12(17.91)	28(10.81)	NS
Acid Regurgitation	22(32.84)	39(15.06)	0.001*
Heartburn	20(29.85)	50(19.31)	NS
Preoperative Ultrasound Diagnosis			
Gallstone Disease			
No. of Gallstones			
Single: Multiple (% Single)	21∶34(38.18)	104∶108(49.06)	NS
Polypoid Lesion			
No. of Polypoid Lesions			
Single: Multiple (% Single)	8∶4(66.67)	18∶31(35.29)	NS
Gallbladder Wall Thickening	12(17.91)	39(15.06)	NS
Atrophic Gallbladder	15(22.39)	35(13.51)	NS
History of Other Gastrointestinal Diseases			
Chronic Gastritis	45(67.16)	124(47.88)	0.005*
Gastric Ulcer	11(16.42)	21(8.11)	0.042▴
Duodenal Ulcer	8(11.94)	12(4.63)	0.026▴
Reflux Esophagitis	12(17.91)	25(9.65)	NS
Chronic Enteritis	3(4.48)	8(3.09)	NS
*H.pylori* (+) in Gastric or Duodenal Mucosa			
Warthin-Starry Stain	39(58.21)	106(40.93)	0.011▴
* H.pylori* Culture16s rRNA gene PCR	33(49.25)42(62.69)	90(34.75)115(44.40)	0.029▴0.008*

* *p*<0.01.

▴ *p*<0.05.

NS: not significant.

N/A: not applicable.

**Figure 5:**
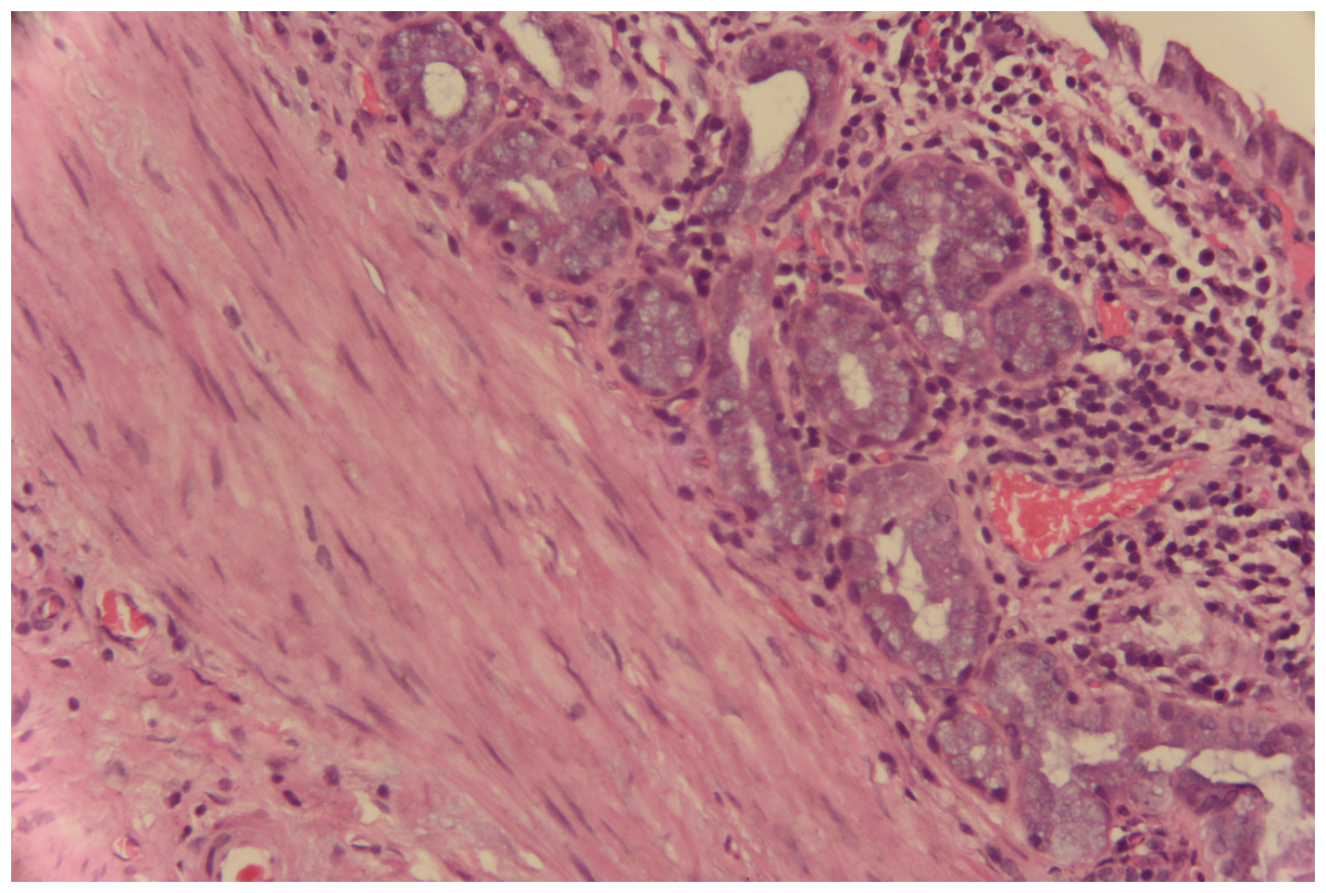
Metaplasia of Chronic Cholecystitis (hematoxylin-eosin stain,×100).

**Figure 6:**
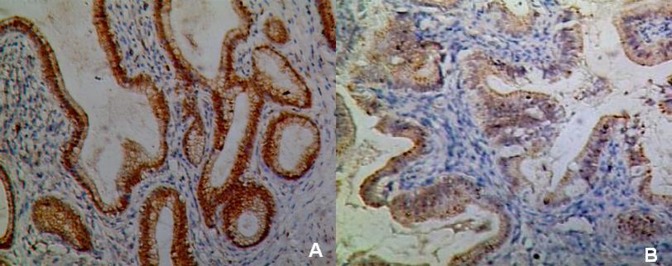
iNOS expression in gallbladder mucosa of chronic cholecystitis with *H.* *pylori* infection (A) and without *H. pylori* infection (B) (×100).

**Figure 7:**
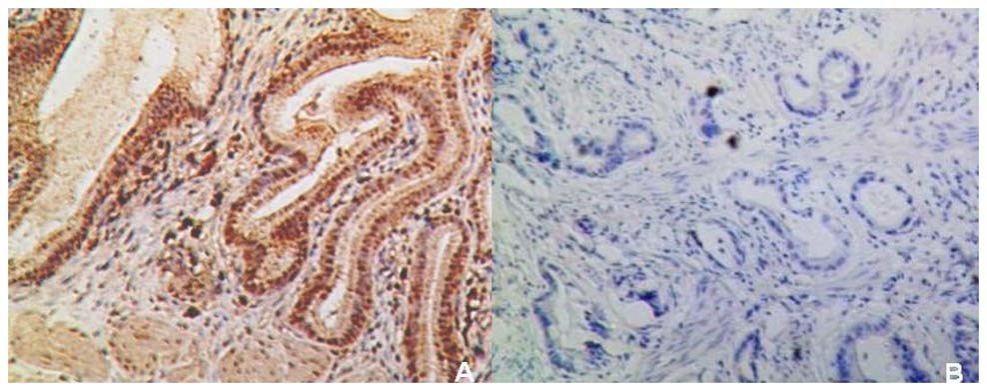
ROS expression in gallbladder mucosa of chronic cholecystitis with *H.* *pylori* infection (A) and without *H. pylori* infection (B) (×100).

The results of comparison of pathological features between the two groups in gallbladder mucosa were demonstrated in Table 3. Higher incidences of adenomyomatosis (*p* = 0.012) and metaplasia (*p* = 0.022) were detected in *H. pylori* infected gallbladder mucosa (Figure 5). The metaplastic lesions were predominantly of pyloric type (21 cases, 84% from the total of metaplastic cases), characterized by structures similar to the pyloric glands in the lamina propria. Intestinal type, which characterized by the presence of goblet cells and enterocitlike cells, was detected in only 16% (4 cases) of all the metaplastic patients. No difference was found in the distribution of the two metaplastic types between the two groups (*p* = 0.602). Regarding iNOS and ROS expression, the immunoreactive scores were both significantly higher in *H. pylori-*positive gallbladder mucosa compared to *H. pylori-*negative mucosa (*p* = 0.012 and 0.000, respectively) (Figure 6 and 7). However, in our study, there were only 3% of the slides showed positive *H. pylori* staining and enhanced iNOS or ROS expressions occurring simultaneously in the same area.

**Table 3:** Pathological characteristics of *H.pylori*-positive and negative chronic cholecystitis.

Characteristics	No. of Patients n(%)		
	*H.pylori* (+) in gallbladder mucosa (n = 67)	*H.pylori* (−) in gallbladder mucosan(n = 259)	*p* value
Postoperative and Pathological Diagnosis			
Gallstone			
Cholesterol Stones	14(25.45)	68(32.08)	NS
Pigment Stones	8(14.55)	35(16.51)	NS
Mixed Stones	33(60.00)	109(51.42)	NS
Polypoid Lesion			
Cholesterol Polyp: Inflammatory Polyp	10∶2	45∶2	NS
Gallbladder Adenomyomatosis	35(52.24)	92(35.52)	0.012▴
Xanthogranulomatous Cholecystitis	5(7.46)	12(4.63)	NS
Histological Analysis of Cholecystitis			
Inflammatory mononuclear infiltrate			NS
Mild	31(46.27)	131(50.58)	
Moderate	22(32.84)	89(34.36)	
Severe	14(20.90)	39(15.06)	
Degree of Fibrosis			NS
Mild	45(67.16)	188(72.59)	
Moderate	14(20.90)	54(20.85)	
Severe	8(11.94)	17(6.56)	
Thickness of Muscular Layer			NS
Mild	30(44.78)	120(46.33)	
Moderate	22(32.84)	103(39.77)	
Severe	15(22.39)	36(13.90)	
Adipose Tissue Deposition			NS
Mild	33(49.25)	166(64.09)	
Moderate	27(40.30)	73(28.19)	
Severe	7(10.45)	20(7.72)	
Degree of Hyperplasia			NS
Diffuse	25(37.31)	115(44.40)	
Focal	42(62.69)	144(55.60)	
Dysplasia			NS
Low-grade	3(4.48)	8(3.09)	
High-grade	0(0.00)	0(0.00)	
Metaplasia	9(13.43)	14(5.41)	0.022▴
Immunoreactive score of iNOS	6.06±1.59	5.12±1.34	0.012▴
Immunoreactive score of ROS	5.01±2.01	3.99±1.87	0.000*

* *p*<0.01,

▴ *p*<0.05.

NS: not significant.

N/A: not applicable.

## Discussion

The presence of *H.pylori* in gallbladder mucosa was first confirmed by Kawaguchi et al in 1996. [18] However, although *H.pylori* has been found 3.5 times more frequently in presence of chronic cholecystitis, whether this agent contributes in the pathogenesis of this biliary disease is still poorly understood. [19] Firstly, it is difficult to verify the potential entry routes of *H.pylori* to the gallbladder including either ascending duodenum infection or the portal system circulation pathway. [20], [21] Secondly, since successful demonstration of *H.pylori* in gallbladder was mostly based on the indirect detection methods such as PCR for *H.pylori-*specific components rather than direct bacterial culture, some investigators believe that *H.pylori* is only a “stagger” but not an “invader” in biliary system. [22], [23].

Consistent with one previous report from Turkey [24] in our study, *H.pylori* was isolated in 20.55% (67/259) of the patients using culture, staining of gallbladder mucosa and PCR for specific 16s rRNA gene. Among the above three techniques, nest PCR still showed the highest sensitivity. We found that *H.pylori* in the stomach was strongly associated with the infection of this bacterium in gallbladder mucosa. Our data also showed a significant correlation between chronic cholecystitis and a few *H.pylori*-related diseases such as chronic gastritis, gastric ulcer and duodenal ulcer. Considering that *H. pylori-*16s rRNA in gallbladder and gastric-duodenal mucosa from the same individual patient had completely identical sequences, we hypothesize that *H.pylori* in the gastrointestinal system might be a potential candidate for increasing the risk of chronic inflammation of the gallbladder. *H. pylori* might reach the biliary system via sphincter of Oddi by the reflux mechanism. Bacteria colonized in the stomach and small intestine in patients with sphincterotomy and biliary enteric anastomoses, recurrent cholangitis and sphincter of Oddi dysfunction might be the cause of secondary gallstones and cholecystitis. [25], [26].

The presenting study revealed that *H.pylori-*infected gallbladder mucosa has a significantly higher prevalence of adenomyomatosis (GAM) than non-infected mucosa (52.24% versus 35.52%, *p* = 0.012). GAM is a benign, degenerative condition characterized by proliferation of the mucosal epithelium and hypertrophy of the muscularis mucosae accompanying with grossly formed mucosal invagination and intramural Rokitansky-Aschoff sinuses. [27] GAM can be diagnosed preoperatively through ultrasound, CT scan or MRI. [28], [29] Incidence rate of GAM is reported to be 25.8%–32% in chronic cholecystitis patients based on the cholecystectomy specimens. [30], [31] Some investigators strongly recommended cholecystectomy in case of GAM with gallstones or symptomatic GAM because stones and chronic inflammation secondary to GAM may lead to dysplasia, metaplasia and cancer. Although no similar finding has been reported with respect to *H.pylori* infection and its association with GAM, we speculate that *H.pylori* might be involved in the development of GAM by altering cell kinetics and proliferative activity which were verified in chronic gastritis and gastric carcinogenesis. [32].

According to literatures, metaplasia of gallbladder mucosa presents in 5%–39% of cholecystectomies. [33]–[35] In our study, metaplasia was identified in 7.67% of the included patients and it was shown to be statistically correlated with *H. pylori* infection in gallbladder mucosa (*p* = 0.047). Metaplasia is believed to be a strong histological sign for diagnosis of moderate or severe chronic cholecystitis because it is rarely observed in gallbladder autospy in which only mild inflammatory changes present. [36] Misra et al. [37] found that *H. pylori* colonises areas of metaplasia in gallbladder producing histological changes very similar to those seen in gastric mucosa. Chen et al. [38] demonstrated that metaplasia may provide suitable conditions for *H. pylori* colonization in the gallbladder. Their electron microscopy revealed at sites infected with *H. pylori*, the integrity of the cell-to-cell membrane of gallbladder epithelium was destructed, with swelling of mitochondria and dilatation of endoplasmic reticulum. In *H.pylori-*infected gallbladder nucosa, metaplasia lesions area accompanying with *H.pylori* colonization could be detected in 91.5% of the specimens. These morphological findings may indicate a potential direction for determining the role of *H.pylori* in the formation of metaplasia. Except metaplasia, hyperplasia and dysplasia were detected in 100% and 3.37% of the included patients, respectively. However, no significant correlation could be set up between these two kinds of pathological changes and *H.pylori* colonization in gallbladder mucosa.

*H.pylori* can damage gastrointestinal epithelial cells through mediating chronic inflammation. In the pathogenesis of gastric cancer, *H.pylori* is proved to promote the expression of ROS/RNS mediated by NF-κB, AP-1 and other pathways. [39], [40] High concentration of NO can lead to nitrative DNA damage and canceration of the epithelium. [41] In our study, the expression levels of ROS and iNOS were significantly increased in *H.pylori* infected gallbladder mucosa than that in non-infected mucosa. However, considering there were only 3% of the slides showed positive *H. pylori* staining and enhanced iNOS or ROS expressions occurring simultaneously in the same area, whether *H. pylori* could directly induce oxidative stress in gallbladder mucosa through increasing the expression of iNOS or ROS in gallbladder mucosa still needs further investigation. Recently, a study *in vitro* showed that *H.pylori* could significantly stimulate the growth of cholangiocarcinoma cell line (KKU-100) and DNA synthesis through iNOS pathway. [42] Unfortunately, no study so far has explored the role of *H.pylori* in the development of chronic cholecystitis in normal gallbladder epithelium with respect to cell proliferation, apoptosis, and inflammation.

### Conclusions

In summary, our study indicated that *Helicobacter pylori* infection in gallbladder mucosa is strongly associated with *Helicobacter pylori* existed in the stomach. *Helicobacter pylori* is also correlated with gallbladder premalignant lesions including metaplasia and adenomyomatosis. The potential mechanism might be related with higher ROS/RNS production in *Helicobacter pylori-*positive gallbladder mucosa.
